# Data-Driven Modeling and Simulation for Optimizing Color in Polycarbonate: The Dominant Role of Processing Speed on Pigment Dispersion and Rheology

**DOI:** 10.3390/ma19020366

**Published:** 2026-01-16

**Authors:** Jamal Al Sadi

**Affiliations:** Faculty of Engineering, Jadara University, Irbid 21110, Jordan; j.alsadi@jadara.edu.jo or jamal.alsadi@ontariotechu.net

**Keywords:** data mining models, opaque PC grades, speed parameters, rheology, DOM, SEM, particle size analyzer, Anova, dispersion, characterization

## Abstract

Maintaining color constancy in polymer extrusion processes is a key difficulty in manufacturing applications, as fluctuations in processing parameters greatly influence pigment dispersion and the quality of the finished product. Preliminary historical data mining analysis was conducted in 2009. This work concentrates on Opaque PC Grade 5, which constituted 2.43% of the pigment; it contained 10 PPH of resin2 with a Melt Flow Index (MFI) of 6.5 g/10 min and 90 PPH of resin1. It also employs a fixed resin composition with an MFI of 25 g/10 min. This research identified the significant processing parameters (PPs) contributing to the lowest color deviation. Interactions between processing parameters, for the same color formulation, were analyzed using statistical methods under various processing conditions. A principle-driven General Trends (GT) diagnostic procedure was applied, wherein each parameter was individually varied across five levels while holding others constant. Particle size distribution (PSD) and colorimetric data (CIE Lab*) were systematically measured and analyzed. To complete this, correlations for the impact of temperature (Temp) on viscosity, particle characteristics, and color quality were studied by characterizing viscosity, Digital Optical Microscopy (DOM), and particle size distribution at various speeds. The samples were characterized for viscosity at three temperatures (230, 255, 280 °C) and particle size distribution at three speeds: 700, 750, 800 rpm. This study investigates particle processing features, such as screw speed and pigment size distribution. The average pigment diameter and the fraction of small particles were influenced by the speed of 700–775 rpm. At 700 rpm, the mean particle size was 2.4 µm, with 61.3% constituting particle numbers. The mean particle size diminished to 2 µm at 775 rpm; however, the particle count proportion escalated to 66% at 800 rpm. This research ultimately quantifies the relative influence of particle size on the reaction, resulting in a color value of 1.36. The mean particle size and particle counts are positively correlated; thus, reduced pigment size at increased speed influences color response and quality. The weighted contributions of the particles, 51.4% at 700 rpm and 48.6% at 800 rpm, substantiate the hypothesis. Further studies will broaden the GT analysis to encompass multi-parameter interactions through design experiments and will test the diagnostic assessment procedure across various polymer grades and colorants to create robust models of prediction for industrial growth. The global quality of mixing polycarbonate compounding constituents ensured consistent and smooth pigment dispersion, minimizing color streaks and resulting in a significant improvement in color matching for opaque grades.

## 1. Introduction

Color mismatches are a serious product quality flaw that cause waste, expense, and sustainability difficulties. It is a challenging, multivariable processing task to achieve uniform color dispersion within the matrix of polymers. Plastic resources production has been an essential industrial subdivision. In 2005, North America sold around 5.0 billion kg of manufactured plastic compounding, equivalent to USD 11.00 billion, despite current commercial pressures [1,2]. Adding to mechanical properties, the color of the sold plastics is an important characteristic. The plastic compounders face a significant dilemma: how to reduce consumption while simultaneously improving the delivery time of their products. This dilemma is especially true for those who offer small amounts to companies working on prototypes, as they often have short lead times [3].

Due to a slight deviation in color, through over-compounding, the whole lot could be rejected. Variations in degradation performance, pigment dispersion or preparations, PP sound effects, etc., are among the many effects that might produce color discrepancies. Popular processing utilities affect the pigments’ ability to disperse in the resin, as well as the rheological properties [4]. Unfortunately, there has been a lack of comprehensive research on the impact of modifying processing parameters, especially with regard to polycarbonates (PCs). Consequently, the impacts of changing PPs on the preferred colorant output are the primary focus of this study.

Shapes made of plastic are often preferred over others. The complex, transparent PC plastic has numerous applications, including outdoor plastics, which can change color drastically under various circumstances; understanding these circumstances is crucial, as is their impact, especially on compounding materials. For a limited range of grade–color pairings, this investigation seeks to learn how PPs impact color matching. In recent years, the plastic industry has focused on understanding the complexities of plastic color matching to produce high-quality products with minimal waste and the right hue. The quantity of light absorbed is clarified to be proportional to the concentration of the absorbing material by Lambert’s law; in contrast, Beer’s law clarifies that the amount absorbed is proportional to the absorbing substance’s thickness [5]. The ideal analytical approach is to use colored plastic from reliable manufacturers to create tiny and medium-sized prototypes utilizing plastic processes. The plant, therefore, receives orders that need to be fulfilled within a few days.

In conclusion, in the absence of absorption and with nearly equal scattering across all visible wavelengths, when the colorant absorbs the observable light, the object is perceived as white [6], which means that the overall amount and kind of dispersion and absorption are ultimately determined by how an item is perceived to be colored. Resins, additives, and pigments make up the three main categories, each of which contains hundreds of individual components. A specific type of plastic is manufactured by combining particular ingredients and additives. The plastic obtains its color from the hues. The pigments absorb intense colors while reflecting others more subtly due to their proportions and shapes. When color assessment in plastic compounding is regulated, the observer and the base of white light—which is created by blending all visible spectrum wavelengths in nearly equal quantities [7]—are replaced with color depth gears, such as a spectrophotometer or colorimeter [8].

Evidence from earlier studies shows that the screw’s speed, feed rate (F-rate), and temperature are significant processing variables that impact the final color features when using modern data analysis techniques, like DTC and OLAP, to analyze data. In general, these elements influence the forces that shear and melting viscosity, which affect the dispersion and redistribution of pigments. These factors also correspond with the morphology of plastic, allowing one to assess the size and count of pigments, which in turn coincide with color clarity. Codes or values are also used to designate colors, enabling more precise color matching—the two data mining techniques utilized. One was OLAP (online analytical processing), and the other was a decision tree classifier (DTC). Online analytical processing (OLAP) helped identify a correlation between variables that caused batches to fail and parameters with significant variance. The DTC was intended to be a decision support tool that could identify combinations of features that could cause color mismatches. In compounded polymers, the DTC investigates features that may lead to color mismatch issues. Data mining and other online analytical processing methods were previously practical to determine these kinds of explanations [9,10]. In this evaluation, the DTC is typically used to investigate possible connections between the components of grade, color, kind, product, and series.

Based on the papers we reviewed, it is evident that no inquiry has utilized the DTC for color mismatch assessment. Some associated manufactured goods (semiconductors) conduct the DTC [11]. Using past data, other researchers have estimated output colors using neural networks [12].

Prior research has found that the ANN method is a practical tool for removing errors from PC color configuration [13] and has an immediate impact on the value of the dE* color data [14]. This experiment utilizes it to minimize errors in the values of the color tristimulus target data (L*, a*, b*). It found that the working conditions and design of the grades’ ultimate screws were very different. Research has shown that the desired color for finishing may be negatively affected by processing circumstances or by solid mixes of resin structure changes and additives [1,15]. At the same time, the distribution of pigments in paint and coloring has been the subject of extensive study compared to plastics, which have received less attention [16,17]. Dispersion mechanisms are significantly different when working with the temperatures, high shear rates, and pressures encountered in industrialized plastics processes [17]. The processing factors’ impact on colorant has been the subject of multiple experiments using compounding [18,19].

Maximize gloss, brilliance, and mix consistency with minimal processing time. Each item would have a maximum loading; too much color is expensive and reduces bearing resistance [20,21]. Increase the mixing time and lower the resin viscosity to improve dispersion and uniformity [22]. Several studies have investigated the impact of processing on mixing in dynamic extrusion through material compounding [23,24]. Many polymer scientists have explored polymer mixing as a crucial subject.

Impact on Pigment Dispersion. The viscosity of the (blend) matrix is one of the significant parameters that could influence pigment dispersion. The viscosity should be low for rapid pigment wetting while high for rapid deagglomeration. Promoting desired outcomes, deagglomeration, and dispersion requires an optimal dispersion; intermediate and sufficient viscosity is needed. Viscosity plays a key role in determining pigment wetting, pigment agglomeration, pigment flow and dispersion, and ultimately, the color shifts. Particle size and color differences are reduced, and a higher peak distribution occurs with increasing the temperature and F-rate. In the case of screw speed, however, the color difference was reduced at the center. In general, the color differences were reduced across different PPs (i.e., °C, rpm, kg/h), and the color output was improved.

Viscosity and Particle Size Distribution. The blend processed in the twin screw caused a lower viscosity; a larger number of particles, e.g., 66%, had a small particle size compared to 61.3% constituting particle numbers at lower speeds (700 rpm) or Temps, which was attributed to the melt’s lower viscosity, causing the break of molecular bonds and the production of a larger number of particles. In addition, the particles could break into smaller sizes due to additives, high pressure, blending, extrusion, injection molding, and shear heating. Higher viscosity was observed in the produced compound, likely due to the smaller particle size distributed throughout the resin matrix. A higher viscous behavior was exhibited by the blend, resulting in the most desired color outcomes. Smaller particle sizes increased the total surface area, leading to higher viscosity; therefore, color matching and degradation will not be affected by a higher shear rate.

As previously noted by Sanchez et al., PC/PBT blends exhibit transparency during the melt stage and solidify into equally miscible solutions [24]. The rheological properties of recycled PCs were studied by Liang and Gupta (2000) [25] and compared with those of a pure PC. Consequently, it is possible to combine the split PC with a pure PC. Its characteristics are unaffected by additions of up to 15% [25]. Rheological and segmental performance of PC/polyester blends were found by Lee S. et al. However, as is typical with all revisions, they demonstrate that the arrangements do not follow the mixing law. Like other studies, they found that the combinations deviate from the mixing instructions [26]. Other scientists studying extruders found that, similar to twin-screw extruders, single-screw extruders could influence dispersive mixing capabilities [27].

Furthermore, a 45 mm diameter, single-screw extruder equipped with eight glass panes was used to examine the color mixing process [28]. Using this extruder, the researchers definitively determined the starting and ending points of color blending. The results showed that mixing quality was directly related to the high processing pressure in the extruder. During the twin-screw compounding period, researchers also examined how torque loading and dispersal performance were influenced by different screw types and operating conditions [29]. The purpose of this research was to analyze the impact of PPs on output color in both isolated and combined trials using a randomized design. The experiments were performed, the PPs were analyzed, and the rheological dispersion was characterized. 

A systematic investigation of resins, additives, and pigments, as well as processing sets and diverse connections, proved the numerical model’s capacity [30,31]. Further, the surface color and appearance variations are comprised in the following research, influenced by changed screw speeds and plasticizer levels, thus highlighting how visible color sound effects are affected by processing speed in extruded PLA [32].

In addition, machine learning was applied to forecast the color processing parameter (CIELAB) values (L*, a*, b*) of extruded thermoplastic resins employing real-time processing data. Their simulation models precisely predict color difference (dE*) between expected and measured values, allowing proactive practice changes to preserve reliable color quality and decrease off-color manufacturing [33].

The research utilized CIELAB color measurements (L*, a*, b*) and dE * to compute the change in color of PLA during twin-screw extrusion at changing temperatures and speed sets. It indicated that greater screw speeds limited dE* and assisted in keeping color accuracy [34].

dE* embodies the color difference in the CIE L*a*b* color space. This is the standard metric for counting the observed difference between two colors.

L* symbolizes lightness (0—black, 100—white);

a* symbolizes the red-green axis (positive—red, negative—green);

b* symbolizes the yellow-blue axis (positive—yellow, negative—blue);

dE* symbolizes color difference.

dL*, da*, db*, and dE* stand for changes in L, a, b, and dE in terms of total color difference.

More specifically, the concerns of scientific researchers about the effects of twin co-rotating screw course processing factors on different grades of the same hue were alleviated.

In earlier historical data mining studies, translucent PC grades were examined, while our analysis is performed in several parts, starting with the opaque grades and divided into the following phases: data mining, PPs, rheological characterization, and dispersions. This study will analyze and correlate the results of data mining and the effect of viscosity, pigment distribution, and processing parameters, and the outcome of the study is to explore the color output results [35,36,37]. Again, in later stages, two grades will be characterized by two processes, the dynamics of pigment distribution and the fundamental thermal–oxidative degradation of the polycarbonate matrix, as the causes of the observed hue shift, especially in yellowness.

Nevertheless, current research frequently modifies several variables at once, making it challenging to pinpoint the precise mechanistic impact of screw speed on distribution quality. There is an absence of a scientific, fundamental understanding of how the pigments’ particle diameter, numbers, and the resulting hue uniformity are directly impacted by speed change independently. Data mining is used to explore the high adjustment mismatching opaque grade color. In this research, for three processing factors across different treatments, a five-level controlled response method was used.

Solution and Contributions

To fill a critical knowledge gap regarding the impact of processing parameters on color quality in polymer systems, this study methodically identifies and assesses the role of processing speed on color discrepancy. In this technique, the processing temperature and feed rate are kept constant while the screw speed is varied over five predetermined levels. Pigment dispersion study based on particle size and particle number histograms, color measurement in the CIELAB color space, and viscosity assessment are some of the micro- and macro-scale experimental approaches used to characterize the material. Furthermore, pigments aggregate distribution as well as dispersion can be directly observed through the use of optical microscopy, which allows for the examination of microstructural characteristics. These structural investigations strengthen the case for a thorough explanation of color discrepancies caused by processing speed variation by connecting the material’s physical morphology to the quantitative findings of particle analysis and colorimetric evaluations.

The goal was to enhance the color quality by determining the rate of processing PARAMETERS that reduce variation in color (dE < 2.0) and evaluate their impacts on viscosity, pigmentation size, and color outcome. This will be stated explicitly in the amended manuscript. According to this, achievement is defined as dE* being less than 2.0. The outcomes, such as the 775 rpm optimal speed that reduces pigment size and lowers dE* to 1.5, will be presented as proof of meeting this requirement. We will additionally state and evaluate the hypothesized relationship between speed, dimension of particles, and dE* in the findings section. Statistical analysis and experimental data mining were carried out to evaluate morphological characterization and rheological properties and to lay the groundwork for suggestions for process improvement; the impact of pigment count percentage, size, and pigment distribution was also investigated. Furthermore, the samples were characterized for the viscosity test and particle size distribution, pigment count percentage, and size at similar temperatures (230, 255, 280 °C) and speeds (700, 750, 800) rpm, respectively. The average particle size for temperatures (230–280 °C) is about equal to 1–3 µm (60–63%) based on the analysis of the results with pigment particle size and count number % measurements (in microns) at three distinct speed and temperature combinations of 700 rpm (2.4 µm) (61.35%), 750 rpm (2.3 µm), and 800 rpm (2.1 µm) (65.5%).

This study utilizes a single, fixed resin formulation. Optimizing particle processing screw speed requires pigment particle speed and distribution data. According to research, screw rates of up to 800 rpm can significantly affect fragmentation. Increasing screw speed changes impact the mean particle size and an extremely small particle fraction, the study stated. Speed may reduce the mean particle size and increase the small particle percentage to 66%. The findings point to a combination of two processes, the dynamics of pigment distribution and the fundamental thermal–oxidative degradation of the polycarbonate matrix, as the cause of the observed hue shift, especially in yellowness. Analyze all parameters to explore the science behind colorant mismatching for opaque color grade. This work offers a targeted framework for optimizing process parameters by providing a novel, correlated understanding of how processing speed directly (1) modifies coloring dispersion (size and number), (2) impacts color metrics, and (3) determines final color quality via microscopic evolution.

## 2. Materials and Methods

### 2.1. Material Formulation

**Figure 1:**
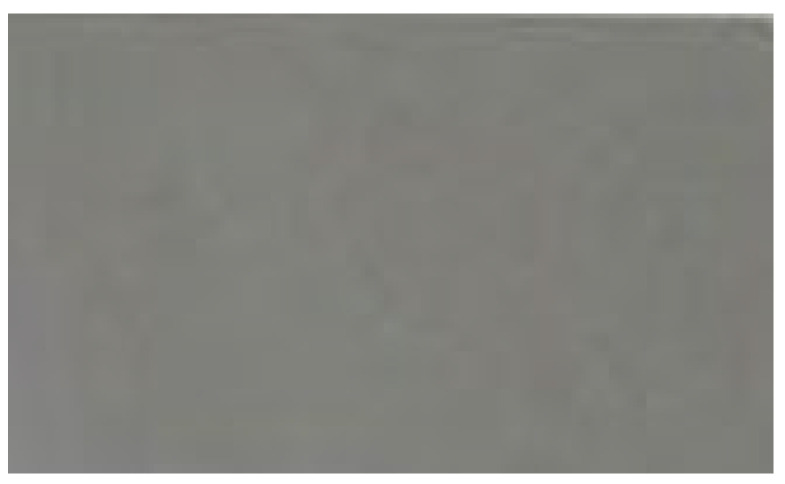
Opaque PC color grade.

Two polycarbonate resins were blended with six different pigments to formulate the opaque color of compounded plastic (Grade 5); this experimental design is based on a constant resin composition, as shown in Table 1 and Figure 1. It contained 10 PPH of resin2 with an MFI of 6.5 g/10 min and 90 PPH of resin1 with an MFI of 25 g/10 min.

This table displays the formulation of a 6 kg batch, which mainly comprises two types of resin, 10% (600 g) and 90% (5400 g), forming the base matrix for the combination. Minor components include six pigments—white, black, green, red, blue, and yellow—each added in small amounts.

### 2.2. Analysis of Particle Size

**Figure 2:**
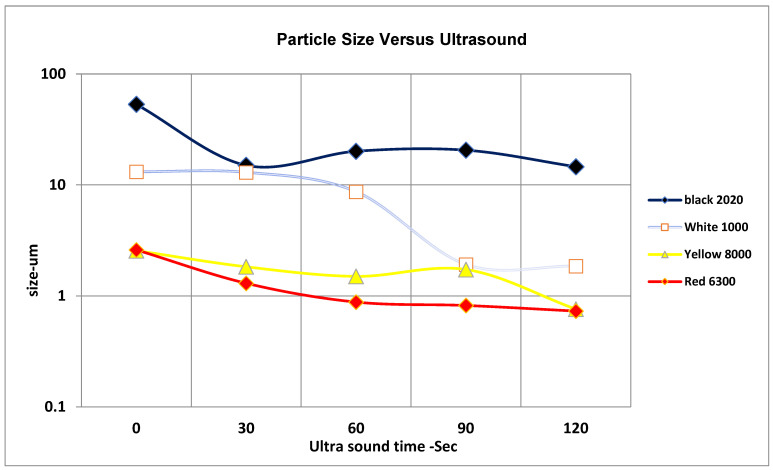
Average particle size for four pigments using wet analysis with varying ultrasound times at 30 W.

In order to assess the size variation among primary pigment samples while they were still wet, a particle size analyzer (PSA) was employed—a Microtrac S3500 (Microtrac Inc., operated with Microtrac FLEX software (version 11) Montgomeryville, PA, USA). Three carefully positioned red lasers were utilized in this model. Diodes that precisely define particles were within a size range of 0.086 µm to 1400 µm. Wet test analysis required the use of recirculation, which included a reservoir for sample introduction, a fluid pump, and a valve for system drainage; the purpose of this was to evenly disseminate the material sample in fluid before delivering it to the analyzer. Triton ×100, a non-ionic surfactant, was added to deionized distilled water with droplets of pigment suspension. This method figures out the size of particles by analyzing the random changes in laser light that are caused by Brownian motion. We look at the particles’ diffusing speed to figure out their hydrodynamic width and dimension dispersion, as shown in Figure 2.

A more recent effort has utilized Dynamic Light Scattering (DLS) for measuring precision at extremely low quantities while improving consistency by taking particle size variations into consideration [38].

### 2.3. Primary Pigment Color

The size of agglomerates and primary pigment particles was successfully decreased by increasing the ultrasonic dispersion power and processing duration.

The following ranges were obtained from an experimental characterization of the pigments’ principal particle size distribution:

Oxide of red iron: 0.8–1.6 µm;
Organic yellow: 0.8–1.8 µm;
White titanium dioxide: 1.7–8.0 µm;
Black channel: 13–20 µm.

The studies revealed that the most essential variables for deagglomerating and reducing the effective particle size of all pigments were raised ultrasonic power and increased sonication time. Thus, the typical primary pigment size is about 1–2 µm, which appears to be somewhat comparable to the particle size of the research pigment.

### 2.4. Spectrophotometer (Measure Color)

A spectrophotometer measures how much light is reflected or transmitted across the visible range to give a color value. It records the precise color coordinates (L*a*b) and the color variance (dE*) compared to a standard. This device gives a number-based evaluation of color that is objective.

This tool is a photometric instrument used to measure spectral reflectance, spectral transmittance, or relative spectral emittance. This study used a CE-7000A spectrophotometer (Konica Minolta, Tokyo, Japan), along with X-Rite Color Master software (version 8.9.6), to quantify the color, and the Standard Observer Function was 1964-10° and D65 light. These pellets were molded using the injection molding machine to produce rectangular color chips (3 × 2 × 0.1″ in dimensions). I maintained injection pressure at about 28 MPa (1000 PSI) and temperature at 280 °C. Color measurements were carried out at three different spots (in each specimen, coupon) to obtain the tristimulus values (L*, a*, b*), where the target values were defined as L* = 63.36, a* −0.34, and b* = 0.20. Then, the color differences were measured as dL*, da*, db*, dE*, and dC*. Studying dispersion using DOM enhances repeatability; it reduces the impact of random measurement error by providing multiple data points per sample.

### 2.5. Data Mining

The study of data mining with an algorithm can identify information quickly about faults related to processing conditions. Additionally, the data for the remaining resins will be studied for faults and correlated with material and processing parameters, and this could help address the issues that lead to improved PPs and total cost reduction.

Several researchers displayed a structured analysis of OLAP integration with NoSQL databases, emphasizing the problems and potential in big data analytics. 

Create a system for supporting decisions utilizing data mining methodologies to improve marketing intelligence and commercial decision-making [39,40].

**Figure 3:**
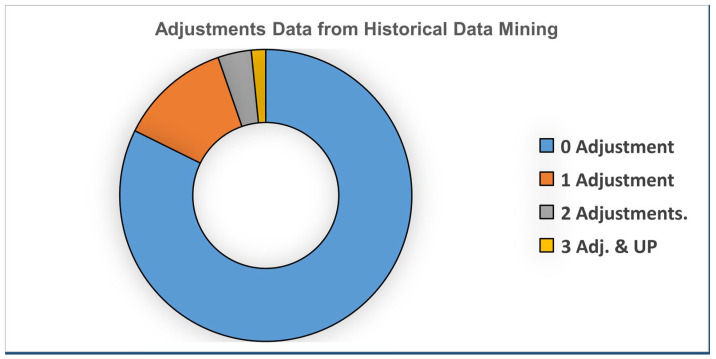
Indicating the percentage of adjustment of the lots during 2009.

The majority of cases (blue) required no adjustment, while a smaller portion needed one adjustment (brown), and even fewer required two or more adjustments (gray and orange), as shown in the pie chart. This indicates a high rate of first-pass success with minimal rework needed (see Figure 3).

### 2.6. A Deeper Window on Data Mining Parameters Investigated

Most companies store their data electronically. This study used data mining techniques to detect patterns among different parameters, analyze, and identify the processed data. The total number of ingredients, pigments, additives, resins (+400), and machinery heterogeneous designs was +15 lines. In 2009, data approaches were used in MS Excel with OLAP to find design improvements for our runs. The reasons for color mismatches and formulations could be better understood if these tendencies in the data could be identified. You may find the outcomes of the data exploration methods mentioned in Table 2. The total number of adjustments is 9598 lots. The required adjustments in the formulations are used more than once, with 17.7%. In this paper, DTC was used for polymer color mismatch cause detection by examining the relationship between different parameters. The polymer parameters studied were color, grade, line, product, and type.

Recent studies utilize the classification of decision trees to ascertain fault sources in high-voltage power lines through RMS-DWT assessment. They introduce a hybrid diagnostic methodology that integrates DTC monitoring with gradient enhancement decision trees for the detection of rotor faults in inducted motors [41,42].

According to the data displayed, only 17.7% of cases required one or more adjustments, with 1.6% requiring three or more, emphasizing that extensive rework is rare, and 82.3% of cases required no adjustment, demonstrating substantial initial precision or quality. Generally, the process looks efficient and well-controlled. In the first nine months of 2009, primary data mining showed that G4 and G5 had the most significant adjustment with red pigment and the same color; 17.7% of the lots were adjusted, as shown in Table 2. The industry standard for high-precision colored work (ΔE* < 1.5), which is used in plastics, paints, and the automotive sector, is an 80–85% first-pass yield. This range signifies the ideal equilibrium between quality control and cost-effectiveness, reflecting a well-regulated process. Attaining an 82.3% yield satisfies these criteria and does not indicate subpar performance; however, it distinctly underscores the potential for optimization, since every modification in the remaining 17.7% substantially escalates the waste of materials, equipment downtime, and total production expenses.

In Table 3, after comparing “good” and “bad” pigments, it appears that the red pigments are the worst because there are only a limited number of good black pigments. After expert analysis, there are two trends.

Letdown pigments (diluted) end in L01. Bad pigments are categorized as black, white, and gray-blue pigments. In the same fashion, it is possible to detect the “good” pigments for the grades.

In this research, Grades G4 and G5 accounted for 9.75% and 2.43%, respectively, in high adjustments, and they had the same color; see Table 3.

## 3. Results and Discussion

### 3.1. Experimentation

Experimentation was conducted at industrial plants (IPs) in Canada using a 27 kW, 25.5 mm German twin-screw extruder, ZSK26, manufactured by Coperion, with a Do/Di ratio of 1.55 and an L/D ratio of 37. Ten heating zones were present, one at the die and nine on the barrel. To feed the material, Brabender utilized a gravimetric feeder (model DDW-H31-FW33-50). The goal color output was a* = 34, b* = 0.17, and L* = 63.38, according to the CIE 1976 standard [43]. For the barrel, PID (Proportional–Integral–Derivative) was regulated with a temperature range of ±0.5 °C, consisting of 9 zones plus 1 die zone. The screw configuration consists of a modular profile with conveying sections, kneading blocks at a 60° stagger, 4 SME mixing elements, a vacuum vent hole at 30D (0.8 bar), and a 4D metering zone. For feeding, a color masterbatch feeder loss of weight (±0.2%) and a base polymer feeder loss of volume (±0.5%) were used. The pre-blending process should take fifteen minutes. The 2 min catch weight test confirmed the calibration. For the stable state, after adjusting, stabilize for 15 min. Sampling started after the melt temperature was stable within 1 degree Celsius and the torque was stable within 3 percent.

### 3.2. Dependence of Viscosity on Temperature (230 °C, 255 °C, 280 °C)

**Figure 4:**
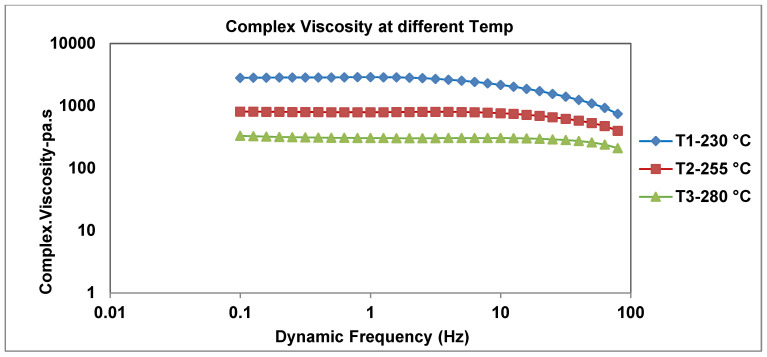
Effect of dynamic frequency at 230 °C, 255 °C, and 280 °C on viscosity for opaque grade.

In Figure 4, as the temperature increases, the viscosity decreases significantly. At 230 °C, the viscosity is high and displays shear thinning performance at high frequencies. At 280 °C, it shows a lower viscosity with less intense changes across frequencies. Figure 4 displays the complex viscosity (Pa·s) vs. dynamic frequency (Hz) at temperatures of 280 °C, 255 °C, and 230 °C. Furthermore, researchers’ analysis shows that the viscosity decreases with rising temperatures. This tendency exposes the communal behavior of polymers, where higher temperatures decrease the viscosity (molecular resistance to flow) due to the properties of shear thinning.

At lower frequencies, the viscosity remains relatively constant across all temperatures compared to higher frequencies (such as those exceeding 10 Hz), particularly at 280 °C, where the viscosity reductions become more pronounced and exhibit the shear thinning characteristic of polymer melts. Additionally, the polymer materials display non-Newtonian behavior. The polymer exhibits temperature-dependent shear thinning behavior, with the maximum resistance to flow at 230 °C and the lowest at 280 °C. Furthermore, some researchers were studying the dynamics of droplets to predict viscosity using machine learning, which is vital for polymer microfluidics. They offer a two-model system to characterize the Arrhenius-like dependency of liquid viscosity [44,45].

### 3.3. Impact of Role Effect of Processing Speed Parameters (Variation Speed, Fixed F-Rate, and Temp)

A precise experiment was performed to study the effects of temperature, screw speed, and F-rate on tristimulus color. The processing elements were well-ordered individually at three different factors while fixing all other parameters (General Trends).

Objective. This research set out to determine how three critical processing variables, speed, F-rate, and temperature, affect the formation of plastic extrusion colors. Its goal was to examine the overarching patterns that emerge from changing these parameters, with an experimental emphasis on separating the effects of rotational speed from those of other variables.

Method. To compound colored PCs, a twin-screw extruder was utilized. Three parameters were tested using a systematic manner; two were kept at fixed, recommended baseline settings, and the third was altered independently over five levels. While maintaining a consistent temperature and F-rate, for instance, speeds ranging from 700 to 800 rpm were examined.

Analysis. A spectrophotometer was used to quantitatively assess the color of the extrudate and obtain CIE L*a*b* values. The extrudate was then injection-molded into test coupons, and the results were analyzed. Thanks to this configuration, we were able to find major interactions between the final color output and the controlled processing conditions.

Based on the observed robust associations, the designated processing speeds were 700, 725, 750, 775, and 800 rpm, with the Temp and F-rate fixed at the middle values (255 °C and 25 kg/h, respectively). Table 4 displays the experimental design (General Trends). Assuming that the variables mentioned above were utilized, in this work, we suggest optimized process speed parameters to attain plastic grade color consistency.

**Figure 5:**
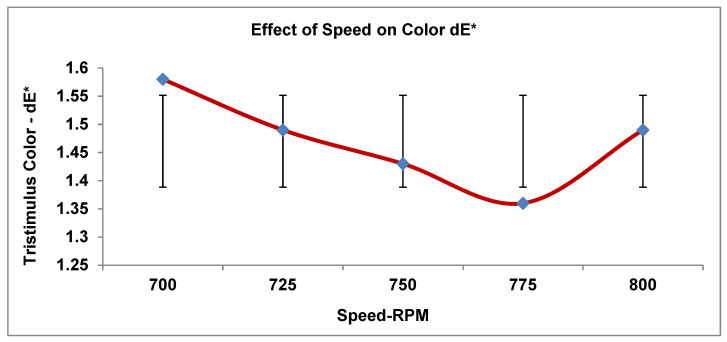
Impact of screw speed on color output.

**Figure 6:**
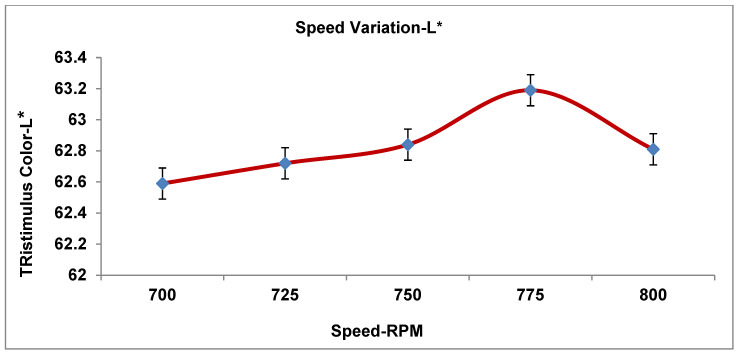
Impact of speed on tristimulus color lightness (L*).

**Figure 7:**
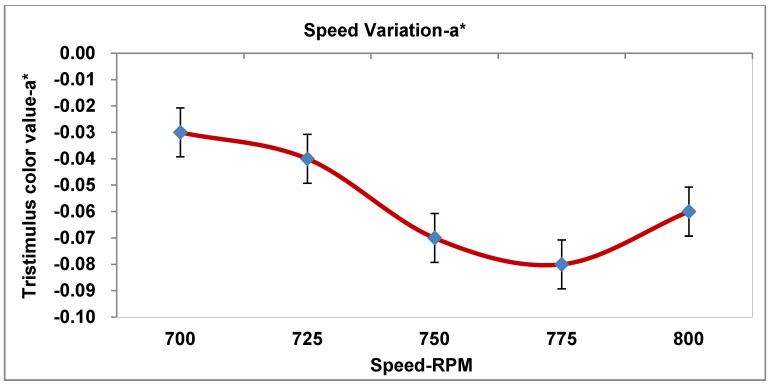
Impact of speed on tristimulus color (redness-greenish—a*).

**Figure 8:**
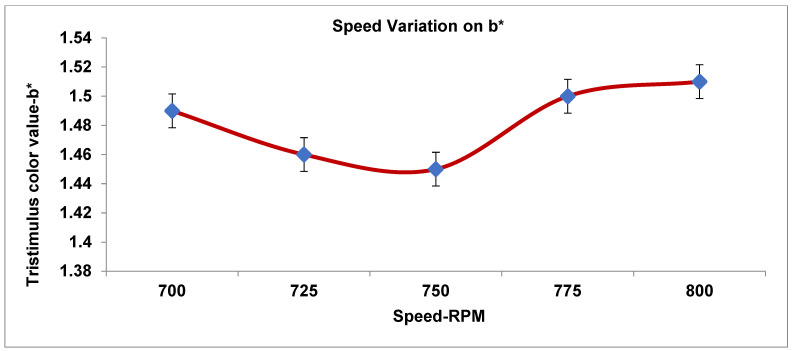
Impact of speed on tristimulus color (yellowish blueness—b*).

**Figure 9:**
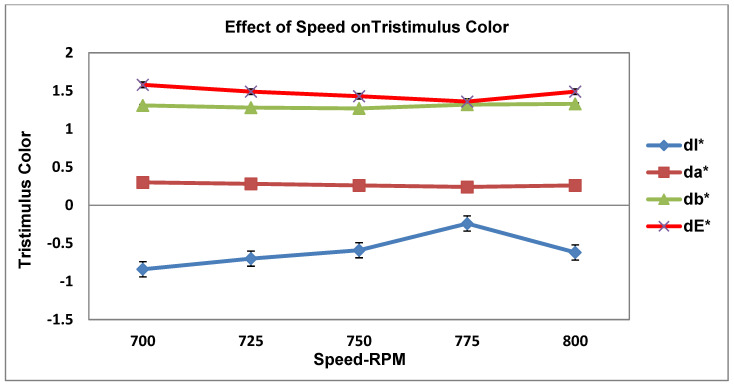
Impact of speed on tristimulus color values.

As recorded in Table 4, the three processing parameters (PPs) were tested in five runs with a total number of design data points for the tristimulus color value. Figure 5, Figure 6, Figure 7, Figure 8 and Figure 9 display the results for the independent variables (CIE dE* values, or deviations from the target) for each of the three PPs as modified across five levels using a specific formulation, opaque Grade G5. The same kinds of graphs were obtained with the CIE tristimulus data (L*, a*, and b*). However, when it comes to color quality dE*, the focus is on trend analysis and dE* values, which show how changing one component affects the other two. The following figures show the results of varying speeds, while the Temp and F-rate were kept constant, which demonstrates the color difference (dE*).

#### 3.3.1. Influence of Fix Temperature (255 °C), F-Rate (25), and Varied Speed Parameters on dE*

Following this, the same process was repeated while varying the screw speed, with the temperature and F-rate held constant. The dE* value drops slightly as speed rises (700 to 800), suggesting that a higher speed slightly improves the color appearances.

From an array of ideal speeds of 700–775 rpm, dE* consistently decreases, indicating improved color precision as speed increases, most likely due to better mixing, dispersion, or decreased residence time. The color value decreased for an arrangement of optimal speeds, at 775 rpm, to its lowest value, as indicated by dE* (1.36), which is the optimal speed value for achieving the color approach to the goal; notably, dE* will increase again as a result of arranging fast speeds of 775–800 rpm. According to Figure 5 and Table 5, color is negatively impacted by reduced contact time, thermal special effects, and/or over-shearing. To achieve this, the speed ending at 775 rpm produces the most accurate color (lowest dE*). When the speed rate is below or above this speed, it results in more deviation from the preferred color.

#### 3.3.2. Influence of Speed on Tristimulus Color Lightness (L*)

The data provided in Figure 6 clarifies the impact of speed variant on tristimulus color lightness (L*) as follows.

Lightness is shown in the CIE (LAB) color matching, where L* expresses the lightness measure when L* equals 0 (which means pure black), but when L* equals 100, it is pure white. However, the middle values between 0 and 100 display varying brightness/lightness values.

For data interpretation observation at 700 to 800 rpm, the results are shown in Figure 6 and Table 6.

(1) 700–775 rpm. Lightness (L*) increases from 62.6 to 63.2, showing an increase in brightness/lightness of the material. This suggests that increasing speed enhances brightness, possibly due to shorter residence time (less thermal degradation) and/or better dispersion or surface finish.
(2) 775–800 rpm. Lightness (L*) drops slightly from 63.2 to 62.8, indicating slight darkening, possibly because of insufficient dispersion at very great speeds or instability of materials at the maximum shear rate. Therefore, the speed obviously affects lightness at a speed of 775 rpm, which indicates the highest lightness (L*).

#### 3.3.3. Influence of Speed on Tristimulus Color on Redness-Greenish (a*)

The absolute value shows the concentration of the red or green color. Wherever a* denotes the red-green axis, a* is greater than zero, which changes the color toward red, and when a* is less than zero, it changes the color toward green. It is observed that the a* values decrease from −0.03 to −0.085, indicating a progressive shift toward green as the screw speed increases. At speeds of 775–800 rpm, the a* tone value rises from −0.085 to −0.06; this is perhaps due to a decrease in green tendency or less time in the shear sector.

The outcome concluded that (a*) illumination shows that all a* values are negative, so all color values stay on the green side of the red-green axis throughout. Additionally, the extreme green concentration occurs at 775 rpm (a* equal to −0.085). Again, after the speed is 775 rpm, the value of green strength drops slightly; at about 800 rpm, it is less greenish than at 775 rpm. The results are shown in Figure 7 and Table 7.

#### 3.3.4. Influence of Speed Variation on Tristimulus Color—Yellowish-Blueness (b*)

The data provided in Figure 8 clarifies the impact of the yellowish-blueness (b*) tristimulus color on the speed variant as follows.

A clear opposite relationship was designated among rotating speed and the b* color assessment; an increase in rotational speed from 700 to 800 rpm, a consistent decline in the b* value (1.52 to 1.40), and a steady and probable change was accompanied by an increase in speed from 750 to 800 rpm as a key control parameter. As a result, a consistent rise (from 1.45 to 1.51) in the b* value and an increase in the material’s yellowness were indicated under fixed conditions; the *b* value shows only a slight, consistent decrease with increasing speed, within a narrow and visually insignificant range in terms of color perception.

#### 3.3.5. Influence of Speed Variation on Tristimulus Color (dl*, da*, db*, dE*)

Figure 9 above displays how changing speed (rpm) affects different tristimulus color parameters.

Consider the following analysis parameters:

1. dL* (lightness). All tendency values are negative, ranging from −0.9 to −0.4. The extruded manufactured goods are steadily darker than the standard ones. dL* comes to be less negative as the speed rises (lightens), where peaking takes place at 775 rpm. Minor darkening occurs again at 800 rpm. In conclusion, screw speed affects the lightness marginally by thoroughgoing lightning around 775 rpm.
2. da* (red-green). The tendency is very steady around approximately +0.3; furthermore, there is no critical difference in color with varying speeds. Therefore, the effect of speed has the least effect on the red-green color trend.
3. db* (yellow-blue). The speed has an insignificant variation with a minor effect on yellow strength.
  The color values are determined to be close to 1.3, with a minor growth and drop.
  The lowest yellow approach is 750–775 rpm, with a minor increase at 800 rpm.
4. dE* (color change). The color has a decrease in value from 1.6 to 1.4 and a variation in speed from 700 to 775 rpm, and its value somewhat increased at 800 rpm; therefore, the best color value of dE* occurs at 775 rpm, which is the most superb operational condition.

### 3.4. Processing Parameters and Experimental Design

#### 3.4.1. Effect of Processing Parameters on Compounding an Opaque Grade (GT)

The effect of processing parameters on opaque and translucent PC compounds was also studied. Of the three PPs, temperature had the greatest effect on color deviation. It was varied at five stages (230–280 °C, 700–800 rpm, 20–30 kg/h), as recorded in Table 4. A decrease in color difference was observed, e.g., from 700 to 775 rpm. These results enable us to focus on speed or temperature for further investigation of its effect on rheology (particularly viscosity) and ultimately on color mismatch. In general, the processing speed was raised to a target range of 775 ± 5% rpm.

#### 3.4.2. Response Surface Methods (DOE): Three-Level Factorial Design

In addition, to reflect the measurement variation, error bars were added to the relevant graphs. In addition, statistical tests (ANOVA) were performed to determine whether the observed differences in dE* values are attributable to chance rather than statistical significance.

Analysis of variance (ANOVA) is essential for validating the significance and fitness of the model. It determines whether the developed quadratic model is meaningful and investigates the effect of the process parameters and their interaction. Methods used include response surface methodology (RSM) with a 3^3^ full factorial design, ANOVA, multiple regression analysis, and desirability function for multi-response optimization. The software used was Design-Expert^®^ V8.0.7.1 (Stat-Ease Inc., Minneapolis, MN, USA). Furthermore, the experimental design included a 27-run full factorial design investigating three factors (Temp: 230–280 °C, speed: 700–800 rpm, F-rate: 20–30 kg/h) with four color responses (L*, a*, b*, dE*).

The same three processing parameters that were studied for General Trends were studied further at the industrial plant. Here, they were subjected to controlled experimentation. Various designs of experiment (DOE) methodologies were employed.

Three essential variables (factors) are tested in this scenario. Three possible values—low, medium, and high—are defined for each. To help you locate the sweet spot, the procedure blends those levels in a predetermined way to simulate their interactions and the impact on your response, as shown in Table 8.

A general factorial design was used in which three levels of each of the three input factors were selected. From the three process parameters, namely, Temp, F-rate, and screw speed (rpm), the experimental design level used is shown in Table 9. 

The suggested 27 combinations of processing conditions for experimentation were simulated using the software package Design-Expert^®^ V8.0.7.1 (Stat-Ease Inc., Minneapolis, MN, USA). Furthermore, the experimental design included a 27-run full factorial design investigating three factors (temperature: 230–280 °C, speed: 700–800 rpm, F-rate: 20–30 kg/h) with four color responses (L*, a*, b*, dE*). This software was used to evaluate the three-level factorial design. The ideal PPs with lower color outcomes had a Temp of 240–250 °C, a speed of 770–790 rpm, an F-rate of 28–30 kg/h, and a predicted dE* of 1.28–1.32.

#### 3.4.3. Analysis of Variance (ANOVA)

The results of a three-level design are analyzed using ANOVA. Significant variation in the response is determined statistically by the factors or their interactions. It is revealed which parameters genuinely influence the process, leading one toward the optimal setting. The experimental results were subjected to ANOVA, as shown in Table 10. Optimization of the processing parameters yielded a set of optimal tristimulus values of L* = 63.36, a* = −0.34, and b* = 0.20. This was well within the range of the maximum allowable deviation of dE* = 2. Speed and Temp had a more substantial impact on color values. The optimized parameters can be used as a baseline for polymer color tests in order to improve the dispersion of pigments.

Furthermore, from the computational analysis of the data, the following results were extracted:

Interpretation of Significance.

1. Temp (A): *p* < 0.0001 (***).
  Extremely significant and explains 45.7% of total variation (0.315/0.690).
2. Speed (B): *p* = 0.022 (*).
  Statistically significant at α = 0.05 and explains 14.1% of total variation.
3. F-rate (C): *p* = 0.011 (*).
  Statistically significant at α = 0.05 and explains 17.2% of total variation.
4. Overall Model: *p* < 0.0001 (***).
  Highly significant and explains 69.9% of total variation (R^2^ = 0.699).

The best settings were 230 °C, 800 rpm, 30 kg/h, and a predicted dE* of 1.317 (close to the best original of 1.235).

The worst settings were 255 °C, 700 rpm, 20 kg/h, and a predicted dE* of 1.517.

#### 3.4.4. Design of Experiments (DOE): Regression Models

Response surface methods (RSMs), such as a three-level full factorial design, were implemented to optimize the process. First, design the experiments and perform them to evaluate the model parameters. Then, for the responses, developing a second-order polynomial mathematical model is needed [46].

(1) $$y=\beta_{0}+\sum_{i=1}^{k}{\;\beta }_{i}x_{i}+\;\sum_{i=1}^{k}\beta_{ii}x_{i}^{2}+\sum_{i}\sum_{j>i}{\;\beta }_{ij}x_{i}x_{j}+\varepsilon \;$$

Y represents the predicted response; β_0_ is a constant; β_i_ is the *i*th linear coefficient; β_ii_ is the *i*th quadratic coefficient; β*_ij_* is the *i*th interaction coefficient; *xi* is the independent variable; k is the number of factors; and ε is the error. When the delta values (i.e., dL*, da*, db*, or dE*) between the target color and the output color are greater than the allowed limits, it is indicated as a color mismatch.

(2) $$dE=\sqrt{(∆{L)}^{2}+({∆a)}^{2}+{\left(∆b\right)}^{2}}$$

That is the Euclidean distance of color deviation in the 3D color space [47]. Customer’s needs dictate the acceptable tolerance limits in units of dL, da, db, or dE.

The quadratic regression model is the starting point with all hypothesized effects.

dE* = 1.415 + 0.011 × A − 0.018 × B − 0.036 × C + 0.098 × A^2^ + 0.016 × B^2^ + 0.020 × C^2^ + 0.058 × AB + 0.043 × AC − 0.024 × BC (3)

The final quadratic regression model is a statistically validated equation that you use for optimization.

dE* = 1.42 − 0.0072 × (Feed − 25) + 0.000157 × (Temp − 255)^2^ (4)

L* = 63.55 − 0.0104 × (Temp − 255) − 0.0022 × (Speed − 750)(5)

a* = 0.026 − 0.00072 × (Temp − 255) − 0.00016 × (Speed − 750) + 0.000038 × (Temp − 255)^2^ (6)

b* = 1.44 − 0.014 × (Feed − 25) + 0.000248 × (Temp − 255) × (Feed − 25)(7)

The recommended optimal compromise is Temp, 240–250 °C, speed, 770–790 rpm,

F-rate, 28–30 kg/h, and predicted dE*, 1.28–1.32.

### 3.5. Determining PSD via Histogram Analysis in Polycarbonate (PC)

Building a histogram with bins representing different ranges of particle dimensions is the first step. We can find the particle dimension frequency distribution by counting the number of pigment particles that fall into each bin’s size interval. Essential characteristics of the dispersion state, such as the mean diameter (and the pigment count number), can be derived from this. One way to describe the quality of polymeric systems’ colors is via this histogram-based PSD investigation. Poor color homogeneity is typically the result of agglomeration or aggregation, which is indicated by the histogram showing a majority of bigger particles.

Stable optical quality and increased mechanical performance are guaranteed by a narrow, evenly spread histogram with consistent bin counts, which reflects a uniform dispersion. Because it establishes a clear relationship between microscopic microstructure and macroscopic color and material qualities, histogram analysis is a simple yet effective approach for assessing pigment dispersion in polymers.

Many researchers compared the additive manufacturing and histogram binning methods for powder PSD measurement, emphasizing particle characterization reliability and precision. This study explores PSD in extremely concentrated polymer dispersions using photon density wave spectroscopy to properly describe particle sizes. It develops PSD-based reactor capacity, polymer quality, and dimension optimization techniques for PVC suspension polymerization [48,49,50].

In this study, histograms are employed to analyze the packaging efficiency of powders with varying particle sizes. The results show that optimized PSD improves flowability and enhances additive manufacturing quality [51].

Measurement reproducibility difficulties are brought to light by histograms of bimodal silica nanoparticles, which show variations among DLS and Transmission Electron Microscopy (TEM) techniques [52].

**Figure 10:**
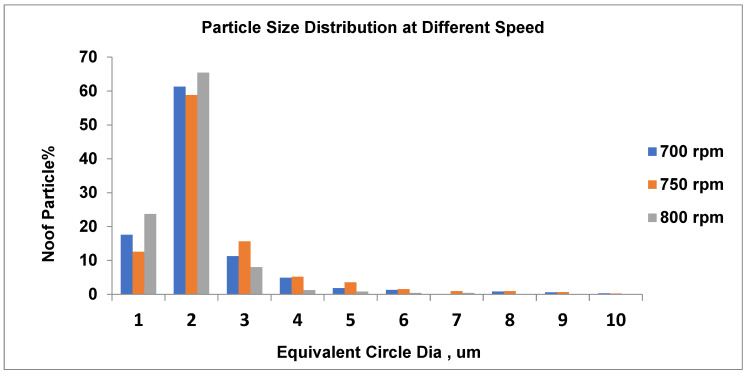
Particle size distribution and number of particles at different speeds.

As shown in Figure 10, at 700 rpm, the number of small particles is around 61.3% (average particle size equal to about 2.4 µm). Furthermore, as the speed rises to 750 rpm, the total number of particles approximately decreases (58.9%) when the average pigment diameter is 2.3 µm. At the highest speed, at 800 rpm, there is a substantial rise in small particle count (65.5%) and a relatively larger average particle size of around 2.1 µm.

At higher speeds, more particles accumulate at smaller diameters, meaning improved breakup and finer dispersion. At lower speeds, the distribution is wider, with more particles at larger diameters, indicating less efficient dispersion. Furthermore, the intermediate speed still peaks at 2.3 µm, but with a slightly wider tail toward larger sizes than the higher speed. According to Figure 4 and Table 5, color is negatively impacted by reduced contact time, thermal special effects, and/or over-shearing. To conclude, the trends indicate that as speed increases, average pigment size decreases, the number of fine pigments increases, and the distribution becomes narrower (improved homogeneity and increased dispersion).

### 3.6. Effect of Speed on Pigment Size (DOM Analysis)

#### 3.6.1. Compounded PC Grade Micrograph (DOM)

**Figure 11:**
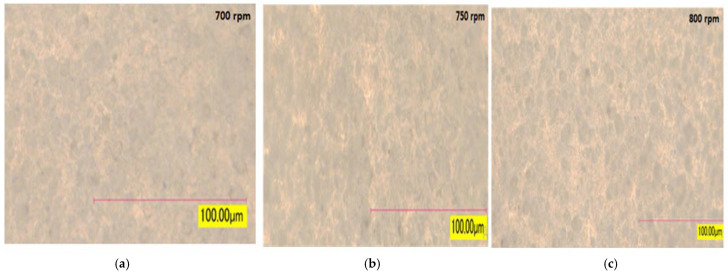
Compounded PC micrograph (DOM) at (**a**) 700 rpm, (**b**) 750 rpm, and (**c**) 800 rpm (scale 100 µm).

Using DOM analysis, the dispersions are examined for different speed samples. Figure 11 shows that the photos were imported into the DOM using color chips at a 100× magnification. In theory, agglomeration can happen in both low- and high-speed zones—the particle size distribution looks relatively identical at a scale of 100 microns. The sample was demonstrated to have good dispersion for consistent color and stable performance. Inconspicuous differences were observed across the three micrographs and compared at 700 rpm, when an uneven texture was exhibited by the surface, revealing noticeable pigment/filler agglomerate, but when the speed was raised to 750 rpm, an improvement in homogeneity was noted, and the clumps were reduced. There are rises in the agglomeration of the particle when the speed rises to 800 rpm due to excess energy, which initially affects the color consequence. The visual image reveals a well-dispersed pigment structure with minimal large-scale agglomeration. The particle size distribution appears relatively uniform at the 100-micron scale.

**Figure 12:**
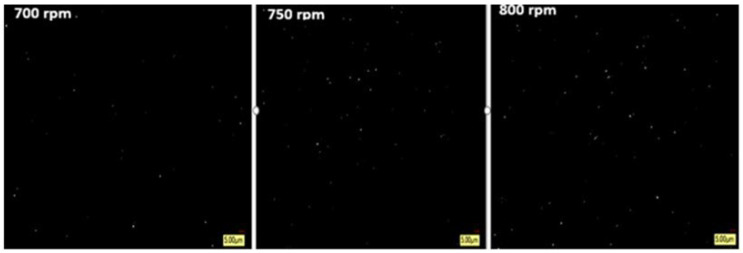
Optical digital microscope at speeds of 700, 750, and 800 rpm (scale 5 μm).

To characterize the advanced diagnostic grade at 5 µm, an additional tester sample was taken, as shown in Figure 12. In this sample, the element size was indicated, as the particle size was observed to be in the few-micron range, which was signified by reasonable dispersion and a rotation speed of 700 rpm. There was very little visible aggregation since the pigment particles were dispersed.

Based on the investigation of Figure 12, the sample treated at 700 rpm exhibits a highly identical and uniform microstructure. At a fine 5 µm scale, a thin distribution of satisfactory, well-dispersed particles is visible, even under dark conditions. This identical dispersion designates that operational homogenization was attained through processing. Additionally, the absence of pigment particle clumping proposes that agglomeration was effectively limited under these specific rotating circumstances.

Based on the analysis of Figure 12, at 750 rpm, with a 5-micron measure, pigment particles have a higher surface area, are effectively processed more, and tend to form weak clusters due to van der Waals or electrostatic forces, preventing whole agglomeration and leading to some gathering. At 100 microns, wholesale things like flowability dictate, but at the 5-micron level, fine-scale interactions affect their behavior, notably providing a more precise and transparent view of fine particle interactions as well as performance.

As shown in Figure 12, compared to the 750 rpm tester, the pigments are less agglomerated and more uniformly dispersed at 800 rpm (particle sizes around 5 microns), preventing the sticking of the particles together to form clumps, which allows for a more uniform distribution due to the increase in the rotating speed. These differences are observed with larger 100-micron particles, which are more likely to produce steady agglomerates at the same speeds.

#### 3.6.2. Particle Size Distribution (PSD) Effects in Opaque Polymer Grades at Three Speeds

**Figure 13:**
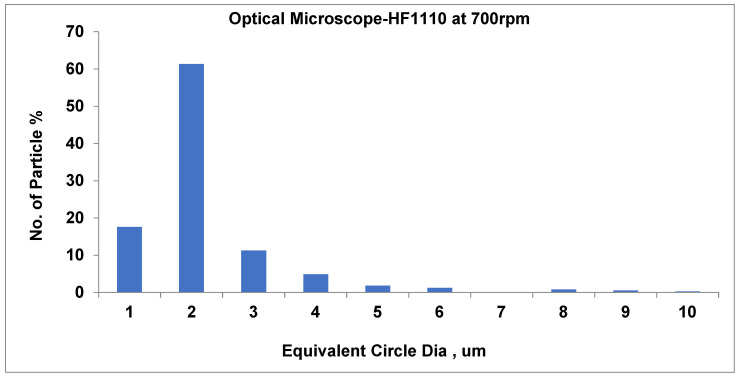
Optical digital microscope at a speed of 700 rpm.

**Figure 14:**
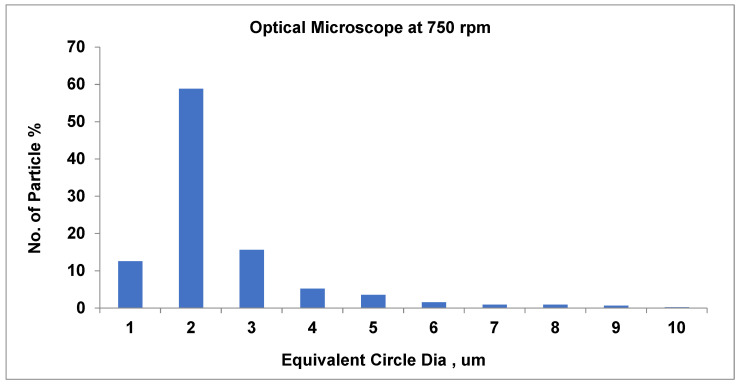
Optical digital microscope at a speed of 750 rpm.

**Figure 15:**
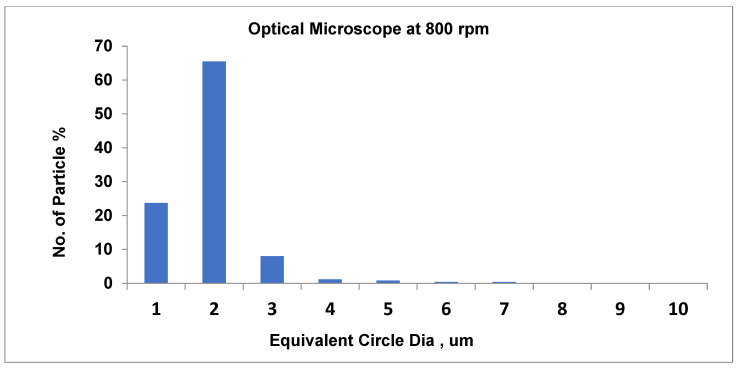
Optical digital microscope at a speed of 800 rpm.

Recent scientists have utilized DEM simulations to demonstrate the impact of PSD form on the behavior of granular and polymeric materials. PSD slope variations are monitored in sediments and are pertinent to polymer composites that interact with the environment [53,54]. The PSD graphs shown in Figure 13, Figure 14 and Figure 15 relate well to the process response values. For 700, 750, and 800 rpm, the average particle size is around 2.2 μm, and the narrowing of the pigment distribution’s highest peak becomes more evident as the screw speed increases. SEM

As displayed in Figure 13, at 700 rpm, the number of small pigments is around 61.32% (the typical average particle size is equal to about 2 µm). Similarly, as shown in Figure 14, at 750 rpm, the pigment constituent amount remains typically acceptable and very well dispersed. The dominant size is about 2 µm, including about 59% of the number of particles; nearly 15% of pigments measure 3 µm, and a lesser number of pigments range up to 10 µm. Agglomeration is negligible, and larger particles seem to be linked to the 700 rpm measurement, while smaller ones are associated with 750 rpm.

To summarize, raising the speed to 750 rpm preserves very good dispersion, but slightly greater energy may cause an insignificant agglomeration of minor particles.

Based on the typical behavior of such processes and compared to the other speed data, the pigment size distribution at 800 rpm indicates a significant shift compared to 700 rpm, indicating that the higher shear force is causing increased aggregation. This reveals that excessive speed is over-processing the mixture, leading to reagglomeration where smaller particles coalesce into larger clusters, thereby worsening the dispersion quality and reducing color effectiveness.

In Figure 15, the diameter of 65.5% of the pigment particles is 2 µm at 800 rpm, which dominates the distribution. Also, the diameter of the majority of particles is 1 µm. Notably, as the diameter increases, the number of particles decreases dramatically, and as the diameter exceeds 3 µm, the number of particles becomes minimal, reflecting effective dispersion and grinding. The majority of particles being concentrated around 2 µm suggests a uniform and well-controlled dispersion process. This size distribution is well suited to achieving stable, high-quality process performance. Based on these analyses, the peaks at 700 rpm are wider at 61.3% and narrower at 65.5% compared to those at 800 rpm. Furthermore, at 700 rpm, pigment particles are very well evenly distributed; they are common within 1–3 µm and dominant within 2 µm. The effective dispersion produced a uniform particle appropriate for steady formulations.

### 3.7. A Scanning Electron Microscope (SEM) Characterization of Raw Color

A scanning electron microscope (SEM) was used. A 20 KV acceleration voltage, a 15 mm working distance, and a 3000× magnification were used with a JSM-600.

To ensure that the four pigments (red, yellow, black, and white) had agglomerates and that primary particles were present in the range of 100 nm, the following was used to analyze the uncoated pigment. 

**Figure 16:**
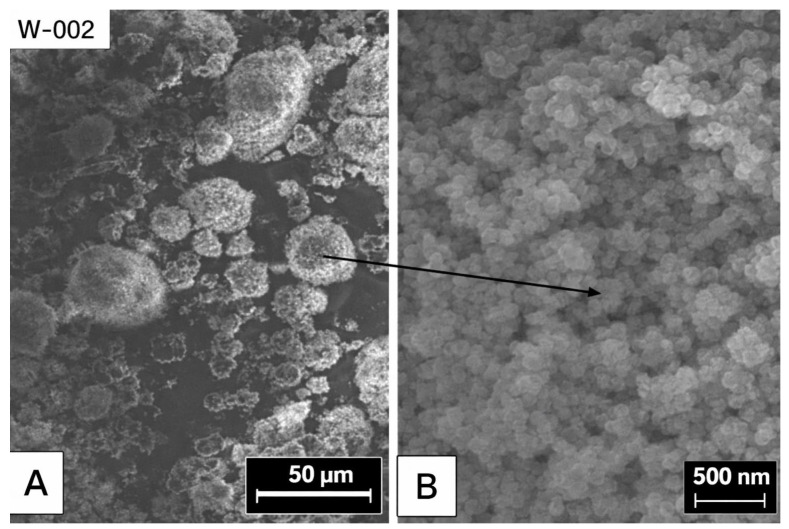
SEM micrograph of white pigments.

**Figure 17:**
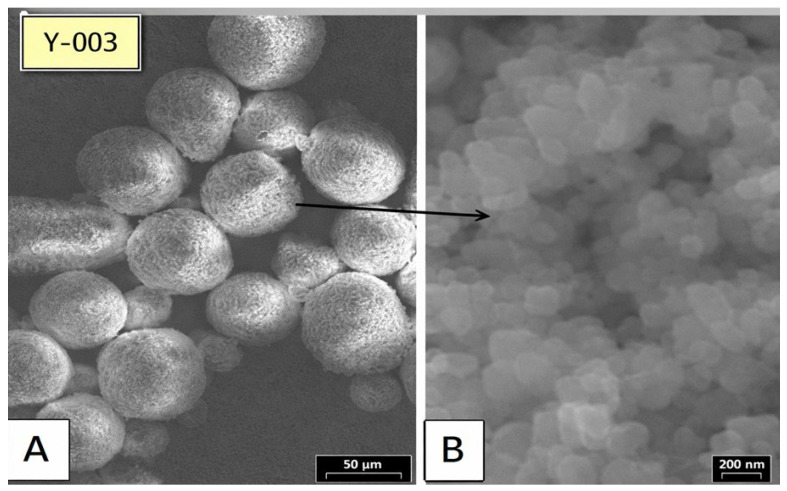
SEM micrograph of yellow pigments.

In Figure 16, an SEM micrograph shows that white pigments contain agglomerates. Primary particles with a spherical shape and a size close to 0.1 μm are shown to exist. Imaging microscopy of yellow pigments is shown in Figure 17. Agglomerates with primary particles that were either elliptical or cylindrical in shape and around 0.1 μm in diameter are seen in the picture. The same holds for the red and black pigments, where agglomerates have spherical primary fragments of 0.1 μm and 10 μm, respectively.

The results from the particle analyzer are comparable to those of the SEM presented in Figure 16 for the primary particle size. The average particle diameter ranged from 100 to 200 nm.

### 3.8. Influence of Viscosity on Pigment Dispersion

The viscosity of the (blend) matrix is one of the significant parameters influencing the pigment dispersion. The viscosity should be low for rapid pigment wetting and high for rapid deagglomeration. This indicates that, for optimal dispersion, there should be an intermediate viscosity that promotes both desired outcomes: dispersion and deagglomeration.

Table 4 illustrates the color outcome in terms of dE*, at the examined speed, and the dE* deviation from the target. The dE* is 1.36 at 775 rpm and 1.49 at 800 rpm, compared with 1.58 at 700 rpm. It illustrates that the viscosity at high shear is quite adequate for wetting the particles.

Utilizing the Washburn equation provided below [55], we can see that the decrease in surface tension and shear viscosity at greater speeds or temperatures is due to increased wetting of pigments.

(8) $$I\left(t\right)=\sqrt{\frac{C\cdot \bar{r}\cdot \gamma_{L}\cdot \cos\theta }{2\cdot \eta }}$$

$I\left(t\right)$, C, $\bar{r}$, $\gamma_{L}$, η, and θ characterize the flow rate of the liquid, the pigment-dependent uniform, average pore diameter of the agglomerate, the surface tension of the fluid, the dynamic viscosity of the fluid, and the pigment fluid contact angle. The rate of pigment wetting is described by the Washburn equation; this rate is influenced by the viscosity and surface tension of the fluid. It connects dispersion, wetting, and viscosity; as wetting improves, the level of penetration grows.

There was cos θ and a bigger pore size (r), which got smaller as the viscosity (η) went up. This means that for best dis-persal and to quickly fill pores, the viscosity needs to be low as well. The Washburn equation states that if the dwell time is too long, the material can become over-dispersed, leading to increased haze and decreased gloss. On the other hand, the pigment will not be sufficiently dispersed if the dwell time is too short; it seems that an energy input is required for dispersion, and this energy is proportional to the surface tension. As a result of lower surface tension, a greater change in surface area occurs for a given amount of dispersion energy, and a lower dispersion energy is required. This is also beneficial for breaking the bonds that hold the primary particles of agglomerates together.

### 3.9. Limitations of the Study

1. Data mining analysis using OLAP and DTC revealed that 17.7% of batches needed changes, successfully identifying difficulties in Grades 5 and 4. However, while the model links process aspects to mismatches, it does not explain the causal significance of formulation discrepancies among these similarly colored classes. Thus, the findings are diagnostic rather than predictive, and they require specific experimental validation before they can be generalized.
2. The study did not independently investigate the impact of temperature-induced viscosity fluctuations (230–280 °C) on PSD and flow, which is a serious limitation. The link between viscosity PSD and color matching (ΔE*) remains unquantified, limiting the thoroughness of the current dispersion model.
3. Parameter-Specific Findings. Grade 5 material with fixed baseline conditions (255 °C, 25 kg/h) yielded the ideal speed range (775–800 rpm) and lowest color difference (ΔE* = 1.36). As a result, these findings may not represent the global optimum, as the diagnostic procedure fails to account for synergistic interactions when all three parameters are modified simultaneously.
4. Limited Material Grade Generalization. The analysis only covers one formulation (Grade 5). As the comparative investigation with Grade 4 (same color, different formulation) is still ongoing, the wider applicability of the observed processing windows to other material systems has yet to be determined.
5. Performance Trade-Off at High-Speed Optimization. While 800 rpm produced the finest mean particle size (2.0 µm) and the highest fraction of fine particles (66%), it also caused particle reagglomeration due to excess shear energy. This illustrates a performance limit, where higher speeds may compromise dispersion quality and color uniformity despite increased main particle size metrics.
6. Constrained Model of Particle–Color Interactions. The investigation shows that reducing mean particle size (from 2.4 to 2.0 µm) and increasing fine particles (from 60% to 66%) leads to better color consistency. The mathematical model that defines how specific PSD changes directly influence the drop in ΔE* remains phenomenological rather than mechanical.
7. Unexplored Synergies and Fixed Parameter Baselines. Temp and F-rate were constant at 255 °C and 25 kg/h, respectively, while speed was changed. Observed synergies, such as lower viscosity from higher temperature, improving dispersion, were not quantitatively investigated inside the speed optimization framework, leaving the combined multi-parameter effects incompletely documented.
8. Future work should, therefore, shift focus from process parameter optimization to material and formulation changes. To fundamentally alter the color development characteristics and enable all color coordinate targets (L, a, b*, dE) to be met simultaneously, investigating alternative pigment grades, stabilizers, or polymer bases is recommended.

## 4. Conclusions

The effect of the PP (General Trends) diagnostic procedure on tristimulus color values was studied for opaque grades. A speed of 775 rpm produced the most accurate color with the lowest dE* (1.36). At this speed, it yields the most accurate color, helping to achieve the optimal speed value to approach the target. More deviation from the preferred color resulted when the speed value was found to be above or below this point. dE* was observed to rise again when reaching a range of 775–800 rpm at the final point. The b* values were stabilized around 1.45, at an F-rate of 25–30 kg/h; however, no further significant improvements were observed. This suggests that a plateau in the yellowness-to-blueness shift has been reached. The lowest dE* (overall color difference) values were at 230 °C and 280 °C, indicating better color match at these extremes. In addition, the result shows that temperature has a slight impact on the overall color difference.

To conclude, there was an effect of PPs on db*. To achieve the aim of improving or minimizing the yellowing color, a medium processing speed is required with a higher frequency, avoiding high temperatures. Furthermore, screw speed affects the lightness (dl*) marginally by thoroughgoing lightening at around 775 rpm.

The reduction in polymer viscosity was achieved by increasing the temperature, particularly at high frequencies, which resulted in an enhancement of shear thinning and flow behavior. The link between speed, mean pigment size, and small particle fraction was found to be positive in this investigation. The mean particle size decreased from 2.4 µm to 2 µm, and the percentage of small particles rose from 60% to 66% as the screw speed went up from 700 to 800 rpm. These findings support the concept that efficient particle size reduction can be improved through increasing the screw speed, which, in turn, produces more microscopic pigments. Further investigation into energy consumption and other factors is needed to optimize processing speed. Supporting the concept are the particles’ weighted contributions, which amount to 51.4% at 700 rpm and 48.6% at 800 rpm. When this model was applied to data collected at various speeds, the lowest color value, dE* = 1.36, was produced at 775 rpm. Enhanced pigment dispersion was achieved by increasing the screw speed, resulting in smaller average particle sizes and narrower particle size distributions. At low to medium speeds, dispersion is less uniform, with larger particle clusters. In contrast, at medium speeds, homogeneity improves, resulting in fewer large particles. Agglomeration, in principle, occurs in zones with both low and high speeds. The microstructure exhibited that the dispersion develops initially at 700–750 rpm. After the optimal speed of 800 rpm, particle agglomeration occurs due to surplus energy, which ultimately affects the color outcome. At 800 rpm, the finest dispersion is achieved, with more small particles and a smooth, uniform surface. Overall, lower viscosity and higher shear (Temp and speed) synergistically enhance color consistency and pigment breakup uniformity. Small particles are generated by the microstructure at high speeds, possessing a higher volume-to-surface area ratio, which leads to stronger van der Waals forces. Therefore, the importance of small particle size was recognized. Furthermore, the metal pigments exhibit a consistent main particle size distribution, ranging from 0.8 to 1.8 µm, as demonstrated by outcomes of the particle size analysis, SEM, and PSD tests. The matching alignment of the opaque grade PSD and the raw pigments SEM test confirms the accuracy and effective dispersion during the given processing circumstances. In the end, based on the DOE and ANOVA outcomes, the designed experimental analysis has identified an optimal operating window for the process. To achieve the target color quality (minimizing the dE* value to approximately 1.28–1.3), which successfully improved the key color difference (dE), the recommended optimal settings are a Temp of 240–250 °C, a screw speed of 770–790 rpm, and an F-rate of 28–30 kg/h. Running the process within these parameter ranges represents the best compromise to consistently produce the desired product quality. As a result, a lower dE* was achieved through the reduction in agglomeration, the presence of smaller particles, and the attainment of uniform dimensions. In conclusion, this research has accomplished significant outcomes by enhancing critical parameters, leading to the maximum level of color excellence.

## Figures and Tables

**Table 1:** Grade 5 color formulation.

	Type	PPH	(For a Batch of 6 kg)
1	Resin	90	5400
2	Resin	10	600
3	Pigment A	1.40044	84.0264
	(White)		
4	Pigment B	0.01051	0.6306
	(Black)		
5	Pigment C	0.03786	2.2716
	(Green)		
6	Pigment D	0.01525	0.915
	(Red)		
7	Pigment E	0.03	1.8
	(Blue)		
8	Pigment F	0.03231	1.9386
	(Yellow)		

**Table 2:** Demonstrating the adjustment (Adj.) of the lots % (2009).

Adj.	# Adj.	% Adj.
0 Adj.	7896.0	82.3
1 Adj.	1186.0	12.4
2 Adj.	358.0	3.7
3 and Up Adj.	158.0	1.6
Total (# of Adj.)	9598.0	100.0

**Table 3:** Different grades adjusted during 2009 had different colors.

#	Grade	Color	% Adjusted Caused by R63100	% Adjusted Caused by R631L0100
G1	MLL4500	70177800	21.95%	
G2	MLL9600	70177800	19.51%	
G3	M14300	5202300		19.44%
G4	M241R00	GY4A15600	9.75%	
G5	MHF11100	GY4A15600	2.43%	
G6	M940A00	70177800	2.43%	

**Table 4:** Fix the two processing parameters and vary the speed (GT).

	Factor 1	Fact 2	Fact 3	Fact 4	Response 1	Resp 2	Resp 3	Resp 4
Run	A: Temp	B: rpm	C: F-rate	D: Grade	dL*	da*	db*	dE*
	°C	RPM	Kg/h					
1	255	700	25	Grade-5	−0.84	0.3	1.31	1.58
2	255	725	25	Grade-5	−0.7	0.28	1.28	1.49
3	255	750	25	Grade-5	−0.59	0.26	1.27	1.43
4	255	775	25	Grade-5	−0.24	0.24	1.32	1.36
5	255	800	25	Grade-5	−0.62	0.26	1.33	1.49

**Table 5:** Trend summary (speed vs. dE*).

Speed (RPM)	dE* Value	Color Precision
700	1.58	Poor (higher deviation)
725	1.49	Slightly improved
750	1.43	Improving
775	1.36	Best color match (lowest dE*)
800	1.49	Color precision decreases

**Table 6:** Perceived color data trend (screw speed—rpm) on L*.

Speed (rpm)	L* Value	Visual Lightness—Weight
700	62.6	Slightly dark
725	62.7	Slight rise in brightness
750	62.8	Brighter/more brilliant
775	63.2	Brightest/most brilliant
800	62.8	Brightness reduces considerably

**Table 7:** Analysis of the effect of speed ranging from 700 to 800 rpm on a*.

Speed rpm	a* Value	Color Shift
700	−0.03	Slight green
725	−0.04	Increasing green
750	−0.07	Stronger green
775	−0.085	Peak green shift
800	−0.06	Green reduces (less intense)

**Table 8:** Parameters and experimental design levels used.

Parameters	Units	3 Levels		
		Low	Medium	High
Temp	°C	230	255	280
Speed	rpm	700	750	800
F-rate	kg/h	20	25	30

**Table 9:** Experimental design (DOE)—three-level factorial design.

	Factor 1	Factor 2	Factor 3	R1	R2	R3	R4
Run	A: Temp	B: speed	C: F-rate	L*	a*	b*	dE*
1	255	750	30	64.01	0.11	1.38	1.38
2	280	750	30	62.96	0.05	1.4	1.28
3	255	800	25	64.22	0.1	1.48	1.6
4	255	700	20	63.73	0.12	1.38	1.45
5	280	750	20	63	0.06	1.44	1.33
6	255	800	30	62.75	0.016	1.29	1.29
7	280	700	25	62.87	0.06	1.5	1.52
8	230	750	30	62.93	0.1	1.37	1.18
9	255	750	20	64.15	0.13	1.46	1.47
10	280	700	20	63.24	0.08	1.4	1.46
11	230	750	20	62.858	0.128	1.52	1.39
12	280	800	30	63.14	0.06	1.44	1.328
13	230	700	20	62.92	0.11	1.38	1.30
14	230	800	30	63.31	0.09	1.4	1.15
15	255	750	25	62.9	−0.086	1.3	1.535
16	230	800	20	63.9	0.038	1.6	1.44
17	255	800	20	61.69	−0.195	1.24	1.67
18	255	700	30	62.05	−0.04	1.21	1.58
19	230	700	25	63.77	0.09	1.48	1.38
20	230	800	25	63.6	0.09	1.375	1.14
21	280	700	30	63.09	0.08	1.42	1.36
22	230	700	30	63.55	0.1	1.39	1.25
23	280	800	25	63.07	0.046	1.45	1.348
24	280	750	25	62.83	0.083	1.47	1.425
25	230	750	25	63.38	0.088	1.475	1.225
26	255	700	25	64.165	0.136	1.44	1.565
27	280	800	20	62.91	0.11	1.4	1.33

**Table 10:** ANOVA summary for color response.

Used Design-Expert V8 Software—ANOVA for Response: dE* (Color Difference)					
Sequential Model Sum of Squares					
Source	Sum of Squares	Mean Square	F-Value Prob > F	*p*-Value	Significance
Model	0.482	0.161	15.42	<0.0001	***
A—Temp	0.315	0.158	15.12	<0.0001	***
B—Speed	0.097	0.049	4.68	0.022	*
C—F-rate	0.119	0.060	5.72	0.011	*
Residual	0.208	0.010			
Lack of Fit	0.096	0.014	1.63	0.252	Not significant
Pure Error	0.112	0.009			
Cor Total	0.690				
Model Summary Statistics					
Std. Dev.	0.1023	R-Squared		0.699	
Mean	1.369	Adjusted R-Squared		0.652	
C.V. %	7.47	Predicted R-Squared		0.571	
PRESS	0.296	Adequate Precision		13.254	

*** *p* < 0.001; * *p* < 0.05.

## Data Availability

The original contributions presented in this study are included in the article. Further inquiries can be directed to the corresponding author.
